# Rabies, with a Report of Two Recent Outbreaks

**Published:** 1898-12

**Authors:** A. B. Kelly

**Affiliations:** Cohoes, N. Y.


THE JOURNAL

OF

COMPARATIVE MEDICINE AND

VETERINARY ARCHIVES.

VOL. XIX. DECEMBER, 1898. No. 12.

### RABIES, WITH A REPORT OF TWO RECENT OUTBREAKS.

By A. B. Kelly, D.V.M.,
COHOES, N. Y.

(Concluded from page 747.)

### Appendix.

### REPORT OF TWO RECENT OUTBREAKS OF RABIES.

### *A. An Outbreak in Greenfield, Saratoga County.*

On June 15, 1897, Dr. T. S. Childs, of Saratoga Springs, was called to the farm of Mr. John Donnon, of Greenfield, Saratoga County, to see a three-year-old grade Jersey heifer which was acting strangely. Upon his arrival he found the patient almost helpless, lying on the floor of her stall. By carefully questioning the owner it was learned that the patient had been bitten by a dog on May 13, 1897, but had shown no signs of ill-health until June 14th, when she was noticed to hook at the other cattle, fences, and trees; stamp her feet, paw, bellow, and refuse food and drink.

The next day I accompanied the veterinarian to the place, but we came to no conclusions, as the animal was in a state of complete paralysis, although the reflex action was present; the eyes were rolling wildly, and the victim was greatly emaciated; temperature 100° F.

After consulting the owner it was decided to dispose of the patient, so, on June 17th, we returned to the place and held a necropsy. Nothing in particular was revealed by the necropsy, except a few small tubercles in the mesenteric glands and lungs and a general congestion of all the mucosæ of the viscera; the brain appeared normal, and the alimentary tract contained very little ingesta. The diagnosis was mesenteric tuberculosis; but, as there was still some doubt as to its correctness, the brain, a section of the spinal cord, a kidney, and sections of the lungs and mesentery were sent to Prof. V. A. Moore for diagnosis. There was some delay in the transportation, and on reaching the veterinary college they were so badly decomposed as to be useless for the desired purpose.

There was considerable controversy in the community as to the nature of the malady, the owner of the cattle and several of his friends believing it was rabies, while the owner of the assailant and the local veterinarian believed firmly that there was no rabies in the county. However, the case was reported to the Commissioner of Agriculture, at Albany, who detailed my brother, Dr. William H. Kelly, of Albany, to investigate it for the State.

On *June* 22*d* we visited Mr. Donnon's place first, and then proceeded to each farm the dog had visited, and, as a result, found that a man named Hart, who lived in Greenfield Centre, owned a shepherd dog that, on May 12th and 13th, ran through the country, acting very strangely, and had attacked three men and several animals. It appears that Mr. Hart formerly lived in Greenfield, but in December, 1895, moved to Greenfield Centre, a distance of about three miles. The dog was always very gentle and friendly, and occasionally returned to his old quarters and visited the neighbors. *May* 12, 1897, he left home and started for another visit, but, when going through Mr. Dean's pasture, he severely bit a fifteen-month-old grade Jersey heifer on the superior portion of the anterior face of the tibial region of the right hind leg, and then attacked Mr. Dean, who avoided a bite by the free use of a club. The dog then hurried on, and went to his old home and bit at the door, but the occupants of the house would not admit him, owing to his queer actions.

He then took the road and ran southward for nearly two miles, attacking every dog he met, and branched off through the fields to the southeast, going directly for Mr. Daniel's farm, where he had a fight with the latter's dog, which appears to be a half-brother to the rabid dog. It was also stated that he bit two hogs on this place, but the owner denied the assertion, and no scars could be found on the alleged victims, which remained perfectly well. After this last encounter he started for home and, as he went across lots most of the way, did not meet any person or animal; he remained home all night.

Early the next morning he bit Mr. Hart's son through his clothing, and started on another journey, going almost due eastward, arriving on the main road, just above Dr. Miller's house; then, starting southward, he met Mr. Donnon, who had known the dog for a long time; but as he had not heard of his strange actions, and did not notice anything queer about the animal, did not try to get out of the dog's way. The brute sprang at Mr. Donnon, grabbing him by his left wrist, but the old gentleman succeeded in freeing himself by choking the dog, but not until his wrist was severely lacerated. When the dog regained his strength he again proceeded southward and turned into Mr. Donnon's farm and bit nearly one-half of the left ear off from a large red cow, and then attacked a three-year-old grade Jersey heifer, lacerating the upper posterior portion of her udder and the inside of the right hind leg. The assailant then started southeast, through the fields, toward Saratoga Springs, but Mr. Hart had been informed of his strange actions, and started out with a horse and wagon to capture the brute. He met the dog near Saratoga and put him into his wagon, but not until he had been severely bitten on the arms and hands; on his arrival home he immediately killed him.

Fortunately for the community, but unfortunately for the authorities' rapid diagnosis, all the dogs bitten by this animal were killed, with one exception, and that was the last dog bitten, belonging to Mr. Daniels. This victim did not contract rabies, due to the fact that he had an exceedingly fine, heavy coat of hair, and that the assailant's teeth were cleansed during his series of fights just prior to this last attack. Mr. Donnon accepted the advice of a Saratoga physician, and on May 16th went to the Pasteur Institute in New York City, received a sixteen-day treatment, and has been in good health ever since.

As was previously stated, Mr. Donnon's heifer was the first animal to show any strange actions. On *July 1st* Mr. Dean reported that his heifer was acting the same as Mr. Donnon's did, so I went to his place and found the animal in a very excitable state. She was tied in a stall by a rope around the horns and, when alone, would stand quietly, with head lowered and eyes half closed; but should a man or an animal come near her she would froth at the mouth, open her eyes, which would have a wild appearance, prick up her ears, attack with her horns, and bite at the rope; temperature 101° F. There were hay and green food in her manger, and she was frequently offered water, once in my presence, but absolutely refused both; patient much emaciated. The wounds were tender, manipulation of them causing increased excitement. The owner was requested to turn her out into a lot adjoining the barn, and the victim at once began to paw, bellow, get up and down, attack the bystanders, and hook at the fence. The salivation was very abundant, and there was a faulty coördination of the muscular movement. A small dog was put in the pasture, and the patient immediately raised her tail, lowered her head for attack, gave a bellow, and started for the canine, chasing him completely out of the pasture.

I stated that it was the most like rabies I ever saw. The next day Dr. Childs, his student, Dr. Kelly, and myself visited the place and found the same condition as on the previous day; but the local veterinarian, being skeptical about the presence of rabies, suggested indigestion, accompanied by foreign bodies in the mouth. Therefore the mouth and larynx were thoroughly searched and the patient treated for indigestion, although no hope of recovery was entertained by the owner or Dr. Kelly. The patient gradually grew worse, the paralysis being more marked daily, and early in the morning of July 5th she died in convulsions.

The brain and a section of the spinal cord were removed and sent in a clean glass jar, containing glycerin, to Prof. V. A. Moore, who received them on July 7th and inoculated two rabbits (Nos. 43 and 44), subdurally, with a suspension of the suspected materials in sterilized water.

These animals remained well until July 29th, when No. 44 was noticed to be partially paralyzed at 7 A.M.; a sharp, loud noise would cause convulsive movements of the head. No. 43 was nervous, but otherwise normal. 10 A.M. No. 44 completely paralyzed; No. 43 partially paralyzed. 5 P.M. No. 44 completely paralyzed; No. 43 better than in the morning.

*July 30th.* No. 44 found dead in the morning. No. 43 partially paralyzed; had convulsive twitchings when disturbed.

*31st.* No. 43 completely paralyzed; remained so during day.

*August 1st.* No. 43 found dead in the morning.

As this was positive proof of the presence of rabies, Prof. Moore sent in his report to that effect. However, there was considerable controversy as to the accuracy of the test, and it went so far that one of the local papers published a long article (author unknown), stating that there was no rabies in the county, and that Mr. Dean was simply scared and starved his heifer to death. This article was contradicted the next week by Dr. Kelly, and the result of Prof. Moore's investigation published.

Neither Mr. Hart nor his family would believe that their dog had rabies, and neither he nor his son paid any particular attention to their wounds; simply kept them cleansed and bandaged until healed. On July 19th Mr. Hart was taken sick, but as he was subject to attacks of catarrh of the stomach he thought little of this attack; but he did not receive the usual relief from the regular remedies, so, on July 21st, he called in his family physician, who at once started to treat him for his old complaint. However, he kept growing worse, had spasms of the glottis, refused food and drink, was very constipated, and died early in the morning of July 22d. The family still scorned the idea of rabies, and the physician, at an interview, said that the man died from gastric catarrh, but also acknowledged that he had never seen a case and did not know the exact nature of rabies. Mr. Hart's son remained well. It was very hard to get this family to talk on the subject, and nothing definite could be learned concerning the man's actions, symptoms, or treatment other than that stated above.

Mr. Daniel's dog showed no ill-health up to this time, but was ordered shut up for six weeks, and has remained well up to the time of writing (April, 1898).

Mr. Donnon's second cow did not contract the disease. No other cases were reported.

B. *An Outbreak of Rabies in Chatham, N. Y.*

On August 15, 1897, a portion of the residents of Chatham, N. Y., were somewhat alarmed over the presence and actions of a strange dog. He was a large black and white dog, with curly hair, and was first noticed in the lower part of the village, running about aimlessly, and did not seem to have perfect control of his hind limbs.

When passing Mr. S. P. Allen's farm the dog attacked a brood-mare in the pasture, but failed in his attempt to bite her. During the afternoon he visited the premises of Mrs. Emily Hurd, and bit her dog, a valuable dashhound, which was playing in the yard. He next bit a fox-terrier belonging to Mr. John Mickle, and immediately afterward bit a bulldog, the property of E. Refenburgh. He next proceeded to Mr. W. H. Barrett's place, and fought with his six-months'-old St. Bernard pup. Mr. Barrett had heard of the assailant's strange actions and accordingly chained his pup to a kennel. All these victims lived in the village, and each one was lacerated principally on the nose.

Continuing his journey, he strolled about two miles southeast of the village, and sprang at the head of a brood-mare owned by Mr. James Doyle, Jr., which was being pastured near the road. The next day it was noticed that she was quite severely bitten on the head and forequarters.

*August 16th.* The same dog was noticed about one and one-half miles further down the same road, in the vicinity of the farms owned by Mr. H. C. Pierson and Mr. John Quirk. He was seen to enter the pastures of each place, but it could not be determined just how many animals he bit. About noon of the same day he was back to the village, chased and bit an English-setter bitch belonging to Dr. H. B. Ambler, and then entered Mr. Elmer Hunt's yard and bit his little dog which was tied to a kennel.

After this attack he returned to the vicinity of Central Square and continued his queer actions, running first in one direction and then in another. Suddenly he uttered a peculiar howl, raised his head high in the air, pulled his tail between his legs, ran up River Street, entered the house of Mr. Benjamin Wilson, and crouched under the table. The occupants were not pleased with their visitor, so they drove him out. During his exit he made a dash at the cat, but she evaded him by climbing a tree. Recognizing his failure to catch the cat, he ran around the house and found a bull-pup tied up. He severely lacerated the pup, disappeared, and was not heard of again that day. No one saw him leave the place, so concluded he had crawled away to die.

The next morning he was found fighting with Mr. C. Wood's shepherd dog; during the day he was recognized at other places on this same road, but no damage was reported until he reached Mr. Fred Wood's place, about three miles east from Chatham. He was lying nearly prostrated on the road near Mr. Wood's house, and the latter's little black-and-tan dog went to investigate. This aroused the rabid animal, and he bit the little dog and proceeded to the adjoining farm, owned by Mr. William Hartigan; had a quarrel with his dog in the door-yard, and went to the pasture, chased and bit at the cows, which in turn bellowed and chased him; but he succeeded in biting some of them.

This was evidently his last assault, for later in the day he was found lying in an old mill-dam in a paralyzed condition. The people threw stones at him, and, after a severe struggle, he dragged himself off into some underbrush, and was left to die. We have no record of his death at this place, but he was not seen or heard of anywhere else, so it is safe to presume that paralysis set in and that he died there.

31*st*. Mr. Refenburgh's bull-dog was taken ill. Symptoms: Refused to eat; deglutition difficult; excessive salivation; paralysis of tongue and lower jaw; later, general paralysis set in, and he died in a few days. He did not bite any person or animal.

*September* 1*st*. John Mickle's fox-terrier was noticed to refuse his food, had trouble with his throat, excessive salivation, paralysis of tongue and lower jaw; was chloroformed.

5*th*. Mr. Hunt's dog refused his food and did not act as usual, so the owner unfastened him from the kennel and started to take him into the house, when he suddenly broke away and ran off, and was found the next day about one-half mile from home in a semi-comatose condition. He also had symptoms similar to those mentioned in the previous cases; dog chloroformed.

5*th*. One of Mr. Quirk's cows, a grade Jersey, was taken sick and supposed to be crazy. Symptoms: Refused food; salivated; yawned; bellowed; chased other cows; sexual excitement; wild expression of face; constipation; milk-secretion completely checked; would make ineffectual attempts to drink; would attack dogs, pigs, and chickens, but did not attack her attendant. Several abrasions, not thoroughly healed, were found on her nose, probably the sequel of the dog's bite. Her eyes were particularly bright and attentive; she appeared to be anxious about something. This condition continued, her voice becoming more hoarse, and she died three days later.

8*th*. Mr. Barrett's pup refused his food and acted strangely, biting at child's clothing; and, thinking it was due to the recent lack of freedom, the owner unchained the pup, which immediately ran away and was not heard of again for four days, when he was found near Garfield, a village thirty miles north of Chatham, in a paralyzed condition. While unfastening the pup, Mr. Barrett's arm was severely lacerated. Mr. Barrett had advertised for the dog, and the finder immediately notified him of the dog's helpless condition. In the meantime he tried to get the dog to take some milk, but he could not, and the victim died the following day. Dog did no damage.

10*th*. One of Mr. Hartigan's cows was taken sick. Symptoms were very similar to those exhibited by Mr. Quirk's cow. She died three days later, and owner held a postmortem and found impaction of the omasum.

13*th*. Another one of Mr. Hartigan's cows began to act the same as the first one. Symptoms throughout the case were the same as those mentioned above. She gradually grew worse, and the owner gave her several drenches of oil to relieve the constipation. One time, when he was drenching the patient, she fell down and broke off a horn. Mr. Hartigan scratched his hands severely on the broken edges, and later put the same hand in her mouth.

This cow died on September 16th. This same day a third cow of Mr. Hartigan's showed symptoms very similar to the other two, except that they were very much milder. Mr. Hartigan began to get frightened when his third cow was taken sick, and he summoned Dr. H. B. Ambler, of Chatham, to his place and related the story to him. After a careful review of the history and examination of the sick cow, the veterinarian diagnosed rabies. Mr. Hartigan consulted his physician and went to Pasteur Institute in New York City, where he received a sixteen-day treatment.

Before Mr. Hartigan's departure Dr. Ambler held a necropsy on the cow that died the previous day, and gave him one-half of the brain to take to New York City with him, and sent the other half, in glycerin, to Prof. Moore, at the New York State Veterinary College.

17*th*. Mr. Wilson brought his bull-pup to Dr. Ambler's hospital for treatment, stating that he had acted strangely for several days. Symptoms: Would wander from home; paralysis of tongue and lower jaw; salivation; priapism; was anxious to drink, but was unable to swallow. Diagnosis, rabies. He kept the dog for observation. During the day the Commissioner of Agriculture, at Albany, was notified of the outbreak, and he sent Prof. V. A. Moore, of Ithaca, and Dr. William H. Kelly, of Albany, to investigate the case for the State. Accordingly, these men arrived in Chatham the next morning, and were met by Dr. Ambler, who assisted with the investigation. Shortly after their arrival the Wilson pup died in paralysis, and Prof. Moore held the autopsy, reserving the brain for examination. The committee then went to Mr. Hartigan's place and saw his cow, which was taken ill two days previously, but they decided to allow her to die of the disease. She died September 22d, but no autopsy was held, as Dr. Ambler was out of town.

During the local veterinarian's absence from town Mr. Doyle's brood-mare was affected. Symptoms: Great excitability; uneasiness; ran a great deal; whinnied; later got down and would bite at the ground and her forelegs. The owner was suspicious of rabies, so destroyed her.

*24th.* Mr. Pierson reported one of his cows as being affected. Patient was a nine-year-old grade Jersey, four months pregnant. The previous night she gave only a small quantity of milk, and the following morning she did not give any. Symptoms: Pulse 60; temperature 103° F.; bellows all the time; saliva running in streams out of each side of her mouth; yawns; refuses food and drink; stands for heifers to ride her; strains but only passes small quantities of feces.

*25th.* Symptoms about the same; she will grab at a stick; chase cats and chickens; appetite depraved, eating dirt, straw and paper in preference to wholesome foods; hyperæsthesia; imperfect coördination of muscular movement when she attempts to run.

*26th.* Symptoms about same, only more advanced. She falls down frequently and bellows loudly. At 2 P.M. she fell down for the last time. She was quite tympanitic; pulse 96; temperature 106.8° F.; respiration rapid; excessive salivation. She was completely covered with flies, but seemed to pay no attention to these, even when they crossed her cornea; strains frequently; opisthotonos present; no passage from bowels; cannot eat or drink anything; died at 6 P.M. The necropsy was held the next morning, and the brain sent to Prof. Moore, wrapped in a cloth which was saturated with a disinfectant.

Upon Mr. Hartigan's arrival at the Pasteur Institute, some guinea-pigs were inoculated with the suspected brain, and all died from rabies. Prof. Moore received the other half of the brain on September 19th, and inoculated rabbits Nos. 52 and 53, subdurally, with an emulsion of the brain in bouillon. Brain had been in glycerin for forty-eight hours. Rabbits remained well for fifteen and twenty-six days, respectively.

*October 4th.* Rabbit No. 53 was partially paralyzed.

*5th.* Rabbit No. 53 was found dead in morning.

*12th.* Rabbit No. 52 was partially paralyzed.

*13th.* Rabbit No. 52 was found dead in morning.

*September 19th.* He inoculated rabbit No. 50 subdurally, and rabbit No. 51 subcutaneously, with an emulsion of the brain from Mr. Wilson's dog.

*October 8th.* Rabbit No. 50 paralyzed in morning, and remained so all day; was found dead the following morning. Rabbit No. 51 did not die from the inoculation.

*September 30th.* He inoculated rabbit No. 54 subdurally, and rabbit No. 55 in the subcutaneous connective tissue of the neck, with an emulsion of the brain from Mr. Pierson's cow. Rabbit No. 55 received two cubic centimetres of the emulsion.

*October* 16*th.* Rabbit No. 54 was partially paralyzed.

17*th.* Rabbit No. 54 was completely paralyzed.

18*th.* Rabbit No. 54 was found dead in the morning.

Rabbit No. 55 did not contract the disease.

9*th.* He inoculated rabbit No. 56 with an emulsion of the brain from rabbit No. 53.

18*th.* Rabbit No. 56 partially paralyzed.

19*th.* Rabbit No. 56 completely paralyzed.

20*th.* Rabbit No. 56 found dead in the morning.

As it has been positively proved that these three animals had rabies, it is safe to presume that all the other animals bitten by that dog, and developing similar symptoms, were affected with the same malady.

All the dogs known to be bitten by this original dog were killed, and no secondary outbreak occurred.

Up to the time of writing (April, 1898), no other cases have been reported in that vicinity.

All the victims were buried. No disinfectants were used in any case.

### References to Literature Consulted.

1. Hydrophobia, M. Pasteur's system, Dr. R. Suzor.
2. Rabies and Hydrophobia, G. Fleming.
3. A Text-book of Bacteriology, Sternberg, p. 6.
4. The Management and Diseases of the Dog, J. Woodruffe Hill, p. 233.
5. Pathology and Therapeutics of the Domestic Animals, Friedberger and Fröhner, vol. ii. p. 585.
6. A System of Medicine, Allbutt, vol. iii. p. 692.
7. A Practice of Medicine, Osler, p. 177.
8. Law. American System of Practical Medicine, Loomis and Thompson, vol. iii. p. 763.
9. Law. American System of Medicine, Pepper, vol. i. p. 901.
10. Moore and Fish. Twelfth and Thirteenth Annual Report Bureau of Animal Industry, 1895-96, p. 266.
11. Diseases of the Dog, Müller—Glass, p. 237.
12. Special Report on Diseases of the Horse, Bureau of Animal Industry, 1890, pp. 215, 526.,
13. Salmon and Smith. Special Report on Diseases of Cattle, Bureau of Animal Industry 1892, p. 396.
14. Manual of Veterinary Microbiology, Mosselman and Lienaux, p. 299.
15. Pasteur and Rabies, T. M. Dolan, M.D.
16. Moore. Cornstalk Disease and Rabies in Cattle, Bulletin No. 10, 1896, U. S. Department of Agriculture, p. 71.
17. Twentieth Century Practice of Medicine, Stedman, vol. xiii. p. 249.
18. Diseases of the Nervous System, Gowers, vol. ii. p. 922.
19. The Principles and Practice of Veterinary Medicine, Williams, p. 224.
20. Nebinger. Hydrophobia, Med. Record, vol. i. p. 532.
21. Adamson. Hydrophobia, Am. Vet. Rev., vol. xix. pp. 45, 147.
22. Lusson. Hydrophobia, Vet. Magazine, vol. i. p. 241.
23. Paige. An Outbreak of Rabies among Cattle, Vet. Mag., vol. ii. p. 34.
24. Butler. Rabies in the Horse, Vet. Mag., vol. ii. p. 193.
25. The Resolutions on Rabies, Vet. Journ., vol. xxiii, p. 54.
26. Penning. Rabies in the Malay Archipelago, Vet. Journ., vol. xxiii. p. 196.
27. Goodall. Rabies and Muzzling, Vet. Journ., vol. xxiii. p. 256.

28. A Singular Case of Rabies in a Dog, Vet. Journ., vol. xix. p. 5.
29. Rabies, Vet. Journ., vol. xix. p. 110.
30. Underhill. A Case of Equine Rabies, p. 214, Vaughan.
31. Canine Rabies in India, JOURNAL OF COMPARATIVE MEDICINE AND VETERINARY ARCHIVES, vol. xvii, p. 782.
32. Clements and Stokes. Rabies in Sheep, JOURNAL OF COMPARATIVE MEDICINE AND VETERINARY ARCHIVES, vol. xviii. p. 271.
33. Bretherton. Hydrophobia in the Horse, Veterinarian, vol. xxxiv. p. 11.
34. Hydrophobia in France and Russia, Veterinarian, vol. xxxv. p. 468.
35. Muzzling of Dogs in Paris, Veterinarian, vol. xxxv. p. 468.
36. Rabies in Cattle, Veterinarian, vol. xliv. p. 723.
37. Earl. Rabies in the Dog, Veterinarian, vol. xliv. pp. 791, 890.
38. Earl. Rabies Among the Albrighton Hounds, Veterinarian, vol. xlv. p. 17.
39. Murray. Rabies in the Horse, Veterinarian, xlv. p. 161.
40. Johnston. Rabies in a Dog, Goat, and two Fowls, Veterinarian, vol. xlvi. p. 760.
41. Spooner. Rabies and Jackals, Veterinarian, xlvi. p. 762.
42. Fleming. The Geographical Extension of Rabies, Veterinarian, vol. xlvii. p. 633.
43. Gordon. Prevention of Rabies and Hydrophobia, Veterinarian, vol. lxiii. p. 380.

